# Deep Neural Network and Radiomics-based Magnetic Resonance Imaging System for Predicting Microvascular Invasion in Hepatocellular Carcinoma

**DOI:** 10.7150/jca.93712

**Published:** 2024-10-14

**Authors:** Zhao-Yi Lin, Kuang Chen, Jia-Rui Chen, Wei-Xiang Chen, Jin-Feng Li, Cheng-Gang Li, Guo-Quan Song, Yan-Zhe Liu, Jin Wang, Rong Liu, Ming-Gen Hu

**Affiliations:** 1 Medical School of Chinese PLA, 100853, China.; 2 Faculty of Hepato-Biliary-Pancreatic Surgery, The First Medical Center of Chinese PLA General Hospital, 100853, China.; 3 Department of Automation, Tsinghua University, 10084, China.; 4 Department of Radiology, The First Medical Center of Chinese PLA General Hospital, 28 Fuxing Road, Beijing, 100853, China.

**Keywords:** Hepatocellular carcinoma, Microvascular invasion, Magnetic resonance imaging, Radiomics

## Abstract

**Background:** Accurate preoperative evaluation of microvascular invasion (MVI) in hepatocellular carcinoma (HCC) is crucial for surgeons to make informed decisions regarding appropriate treatment strategies. However, it continues to pose a significant challenge for radiologists. The integration of computer-aided diagnosis utilizing deep learning technology emerges as a promising approach to enhance the prediction accuracy.

**Methods:** This experiment incorporated magnetic resonance imaging (MRI) scans with six different sequences. After a cross-sequence registration preprocess, a deep neural network was employed for the segmentation of hepatocellular carcinoma. The final prediction model was constructed by combining radiomics features with clinical features. The selection of clinical features for the final model was determined through univariate analysis.

**Results:** In this study, we analyzed MRI scans obtained from a cohort of 420 patients diagnosed with HCC. Among them, 140 cases exhibited MVI, while the remaining 280 cases comprised the non-MVI group. The radiomics features demonstrated strong predictive capability for MVI. By extracting radiomic features from each MRI sequence and subsequently integrating them, we achieved the highest area under the curve (AUC) value of 0.794±0.033. Specifically, for tumor sizes ranging from 3 to 5 cm, the AUC reached 0.860±0.065.

**Conclusions:** In this study, we present a fully automatic system for predicting MVI in HCC based on preoperative MRI. Our approach leverages the fusion of radiomics and clinical features to achieve accurate MVI prediction. The system demonstrates robust performance in predicting MVI, particularly in the 3-5 cm tumor group.

## 1. Introduction

Hepatocellular carcinoma (HCC) is a prevalent primary liver malignancy, necessitating effective treatment strategies such as surgical resection and liver transplantation^1^-^3^. However, HCC exhibits high postoperative recurrence rates, reaching approximately 70% after surgical resection and 35% after liver transplantation^4^. Microvascular invasion (MVI) has been confirmed as an independent risk factor contributing to tumor recurrence and metastasis following liver resection in HCC patients^5^. Thus, precise preoperative evaluation of MVI in liver cancer facilitates informed treatment decisions by surgeons. Patients at high risk of MVI should undergo Chouinard segment-based anatomical liver resection to minimize the recurrence rate. HCC patients undergoing liver transplantation exhibit improved prognoses when MVI is absent^6^. Histopathological examination remains the current diagnostic standard for MVI. However, the intratumoral heterogeneity that results in sampling errors and potential implant metastasis renders preoperative biopsy detection of MVI unfeasible. While the morphological characteristics of tumors can aid in MVI prediction, the definition of these characteristics primarily relies on subjective reader judgment and lacks objective and quantitative indicators. Gradually, magnetic resonance imaging (MRI) examinations have been employed for preoperative MVI prediction due to their ability to provide soft tissue contrast, reflect tumor-related changes in blood flow, offer multiple sequences, and avoid radiation exposure. While the morphological characteristics of tumors can aid in MVI prediction, the definition of these characteristics primarily relies on subjective reader judgment and lacks objective and quantitative indicators.

Radiomics is a quantitative approach to the description of medical imaging. While lacking a strict definition, radiomics aims to quantitatively extract replicable information, including measures of heterogeneity and shape, from diagnostic images. It can be utilized independently or in conjunction with demographic, histological, genomic, or proteomic data to address diverse clinical challenges 7. Computer-aided diagnosis (CAD) technology has gained widespread use in radiomics. Currently, the foremost technology for computer-aided diagnosis and treatment is deep learning, which employs large-scale datasets to construct deep neural networks^8^. Deep learning has demonstrated comparable accuracy to radiologists in diagnosing and analyzing survival outcomes in various diseases, including lung cancer, skin cancer, and breast cancer^9^-^14^. In recent studies, Wu.^15^ employed deep learning techniques to predict the presence of MVI in medical imaging with promising results. However, their study was limited to a relatively small cohort of 117 cases, and the network training process required manual cropping of tumor areas. Similarly, Nebbia *et al.* 16 achieved a notable area under the curve (AUC) of 0.8669 in MVI prediction using five different MRI sequences. Nevertheless, this approach also relied on manual marking of the tumor area.

To address these limitations, this article introduces a novel deep learning-based method for fully automated MVI prediction. The proposed method combines radiomics and clinical features to enhance prediction accuracy and provide valuable guidance for surgeons in selecting appropriate surgical plans and postoperative treatments.

## Materials and Methods

### Patients

In this study, we conducted a retrospective analysis of patients who underwent liver cancer treatment at our hospital between January 2018 and June 2020. The inclusion criteria encompassed patients who met the following conditions: (1) no prior treatment for hepatocellular carcinoma, (2) underwent contrast-enhanced liver MRI examination within one month before surgery, with images meeting the required standards for evaluation, (3) absence of extrahepatic metastasis according to preoperative evaluation, and (4) availability of complete clinical features within seven days before surgery and postoperative pathology reports. The diagnosis of MVI was based on the Sumie standard^17^. Patients meeting any of the following criteria were excluded: (1) a prior history of malignancy, (2) identification of tumor thrombus in hepatic vessels through MRI examination, and (3) incomplete availability of clinical features.

In this study, we employed a rigorous search strategy to identify and enroll a cohort of 420 consecutive patients, with 140 patients allocated to the microvascular invasion (MVI) group and 280 patients assigned to the non-MVI group. Given the limited nature of the available data, we adopted a robust methodology to ensure reliable results. Specifically, we performed 20 independent tests, with each test employing a 3:1:4 random division of the dataset into training, validation, and test cohorts, respectively. The training cohorts were utilized for model training, the validation cohorts were employed to optimize hyper-parameters, such as different sequences or sequence fusion, and the test cohorts were used to evaluate the final performance metrics of our proposed method.

### Pipeline of the Automatic System

**Figure 1:**
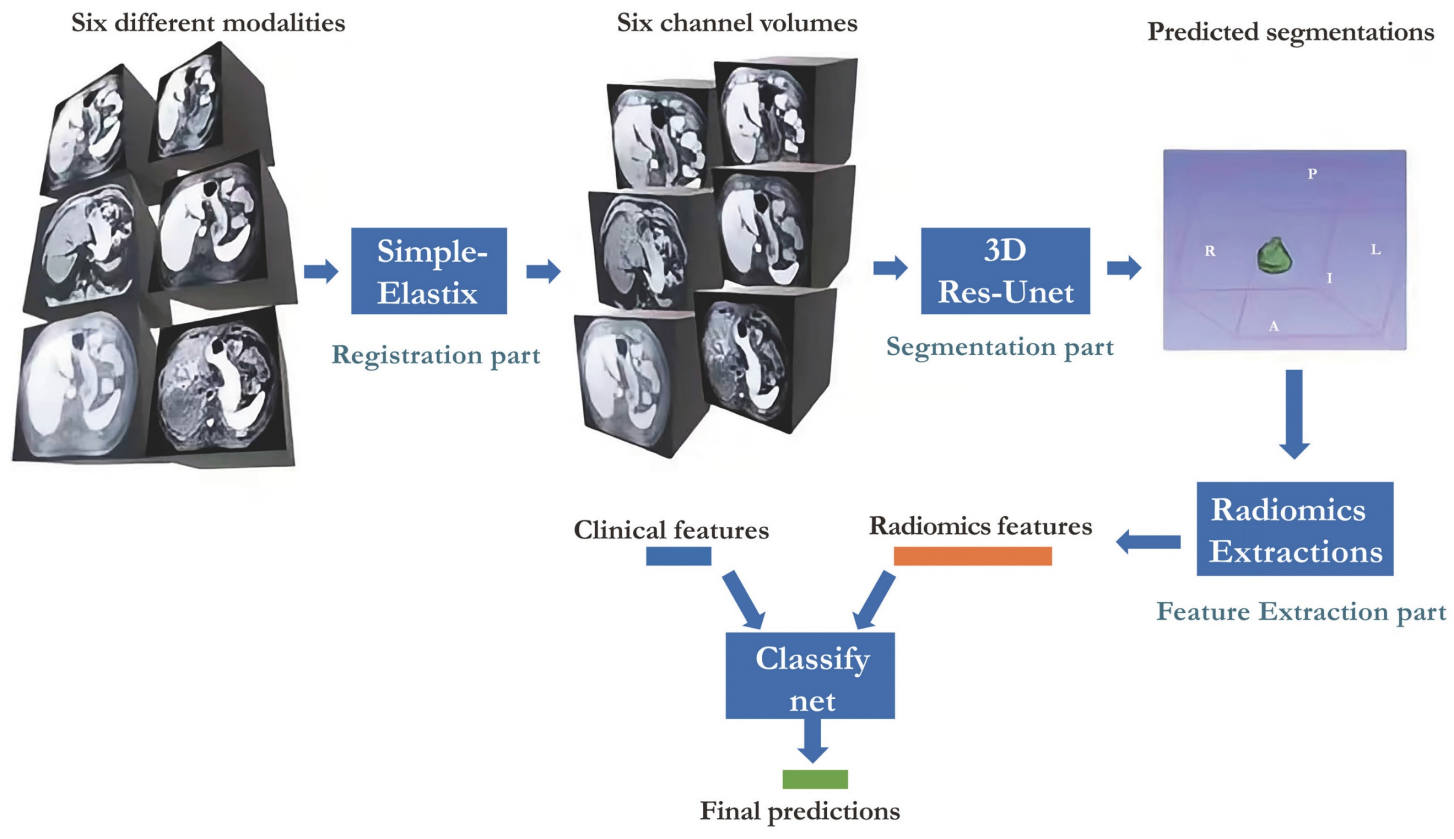
Workflow of the whole system proposed.

Our study presented in Figure 1 illustrates the comprehensive approach we employed, encompassing registration, segmentation, feature extraction, and classification. Our neural network builds upon the Res-Unet architecture, a modified version of Unet specifically designed for this purpose^18^, ^19^. Notably, we successfully automated the identification of HCC-related areas, a task that previously consumed significant time in radiomics analysis studies. This streamlined end-to-end training process is widely regarded as a superior solution.

### Radiology Protocols

MRI examinations were conducted using a 3.0 T MR system (Discovery 750W, General Electric Company, America) in accordance with established protocols. The imaging parameters for the six distinct sequences employed are provided below:

T2-weighted imaging (T2-WI): TR = 12000 ms, TE = 90 ms, slice thickness = 7 mm, Voxel Size = 1.76 × 1.32×7.00 mm, FOV = 38.0 cm, matrix = 256 × 256

Diffusion-weighted imaging (DWI): TR = 5000 ms, TE = 56.1 ms, slice thickness = 7 mm, Voxel Size = 2.97 × 2.97×7.00 mm, FOV = 38.0 cm, matrix = 256 × 256

T1-weighted imaging (T1-WI) (pre-contrast): TR = 3.7 ms, TE = 1.1 ms, slice thickness = 7 mm, Voxel Size = 1.98 × 1.56×5.00 mm, FOV = 38.0 cm, matrix = 256×256

T1-WI (arterial phase): TR = 2.8 msec, TE = 1.3 msec, slice thickness= 7 mm, Voxel Size = 1.98 × 1.56×5.00 mm, FOV = 40.0 cm, matrix = 256 × 256

T1-WI (portal phase): TR = 2.8 msec, TE = 1.3 msec, slice thickness = 7 mm, Voxel Size = 1.98 × 1.56×5.00 mm, FOV = 40.0 cm, matrix = 256 × 256

T1-WI (hepatobiliary phase): TR = 2.8 msec, TE = 1.3 msec, slice thickness = 7 mm, Voxel Size = 1.98 × 1.56×5.00 mm, FOV = 40.0 cm, matrix = 256 × 256

During the examination, a dosage of 0.2 mmol/kg of Gadoxetic acid disodium (GD-EOB-DTPA) was administered intravenously at a rate of 1.5 ml/s.

### Clinical Features

Each patient underwent liver function test, blood routine examination and coagulation function test within 7 days before surgery. Additionally, serum tumor marker tests were conducted, encompassing α-fetoprotein (AFP), carcinoma embryonic antigen (CEA), CA125, CA15-3, CA724, and CA19-9. Screening for hepatitis was performed through the measurement of hepatitis B surface antigen (HBsAg), hepatitis B surface antibody (HBsAb), hepatitis B e antibody (HBeAb), hepatitis B e antigen (HBeAg), hepatitis B core antibody (HBcAb), and hepatitis C antibody (HCVAb). Tumor characteristics were assessed using MRI, where the maximum diameter and number of tumors were quantified.

### Image Annotations for HCC-related Areas

Although our approach was primarily automated, manual annotations were still required to train our deep neural networks and assess the performance of our method. To obtain accurate annotations for HCC-related regions, we engaged the expertise of two experienced radiologists (LJ and SG) with 15 years of experience in the field. A research assistant then verified the consistency of the segmentations, and experienced radiologists reviewed and refined the final segmentation. The HCC-related areas were visualized using MRI. Given the presence of six different sequences that were not aligned, each radiologist was tasked with annotating three distinct sequences: T1-WI pre-contrast, T1-WI hepatobiliary phase, and T2-WI. The margins of HCC in DWI were often indistinct; therefore, annotations were not performed on this sequence. T1-WI pre-contrast and T1-WI hepatobiliary phase represented the beginning and end time points of the T1-WI imaging, respectively, during which patients may have changed positions. T2-WI sequences typically had different spacing from T1-WI, necessitating the annotation of T2-WI. The radiologists employed 3D slicer (version 4.10.1) to generate all segmentation marks. As other methods commonly rely on manual annotations for radiomics, these annotations were also utilized to extract radiomics features. In subsequent experiments, we compared the results obtained using our proposed pipeline with those obtained when the area was manually annotated.

### Registration for Alignment between Multi Sequences

In this study, all patients underwent multi-sequence MRI scans within a brief time interval of approximately 30 minutes. Despite the majority of patients remaining motionless during the scans. We aimed to address the potential misalignment issue in multi-sequence MRI scans. In this study, we employed the SimpleElastix tool for initial alignment^20^.

Following the alignment process, we performed rigid registration between the T1-weighted imaging (hepatobiliary phase) and all other sequences except it. This resulted in a nearly aligned set of sequences. To evaluate the registration performance, we utilized annotations derived from the same registration transforms. Specifically, the tumor areas in T1-WI (pre-contrast), T1-WI (hepatobiliary phase), and T2-WI were expected to overlap with each other. The Dice index, calculated as the intersectional area divided by the sum of both areas, served as the metric for measuring the matching score of different sequences. A higher Dice index following the registration process indicated improved matching between sequences, this step was shown to be beneficial.

### Segmentation for Automatic HCC-related Area's segmentation

In this study, we employed a Res-Unet-based deep neural network to automate the segmentation of HCC-related areas. Traditionally, these areas were manually delineated in radiomics analyses^18^, ^21^. Convolution and pooling processes were employed to extract deeper and more abstract features from the encoded images in the neural network. The application of deconvolutions and upsampling processes facilitates the reversal of the encoding process, yielding features or results with higher resolutions. By employing both approaches, the structure of Unet resembles the letter "U," enabling effective connections between the encoding and decoding parts, thus enhancing the overall outcome. Res-Unet employed Res-Blocks as replacements for all the convolutional blocks in Unet. Considering the presence of 6 sequences, the network's input module was updated to accommodate 6-channel input. The deep model was implemented using the Python programming language and the PyTorch package.

The images underwent standardization, a process of remapping them into a suitable gray value range (0 to 1) required by the deep neural network. Patch-based training and inference were employed for segmentation networks due to the GPU's inability to process the entire volume. During training, the patches were sampled with dimensions of (64, 128, 128) and a stride of (10, 20, 20). However, during testing, the stride was adjusted to (32, 100, 100) to expedite processing time. The training process involved 2000 steps with a learning rate of 0.0002, resulting in an approximate duration of 6 hours. The segmentation performance was evaluated using the Dice-index, comparing manual annotations with automatic segmentation.

### Radiomics Features Extractions

Radiomics involves the analysis of image segments using manually defined statistical features, which are extracted using the Pyradiomics library^7^.

The radiomics analysis encompasses a comprehensive set of features, including 3D-shape features, First Order Statistics, Gray Level Cooccurrence Matrix (GLCM), Gray Level Run Length Matrix (GLRLM), Gray Level Size Zone Matrix (GLSZM), Gray Level Dependence Matrix (GLDM), and Neighboring Gray Tone Difference Matrix (NGTDM). Based on the study conducted by Nebbia *et al.* 16, it was determined that the margin plays a significant role in predicting MVI. In our experiments, we incorporated this finding by extracting margin radiomics for each sequence. Unlike Nebbia *et al.* 16, who did not extract features from augmented images, such as wavelet-transform images and Laplacian-of-Gaussian images. However, our belief was that extracting as much information as possible would lead to a closer fit of the model; thus, we proceeded to extract all available radiomics features, resulting in14736-dimensional (14736-d) features for each multi-sequence volume. For dimensionality reduction, we employed Principal Component Analysis (PCA) on the features, resulting in a transformation to a 20-dimensional representation. The PCA parameters were derived from the training cohort and directly applied to the validation and test cohorts without additional fitting.

### Final Classification Layer and Univariate Analysis

In our study, we utilized a two-layer fully connected network for conducting multivariate analysis. To enhance the outcomes, we incorporated a Relu layer and Batch-normalization. The fusion of these features occurred in the second layer, and to mitigate overfitting, we employed dropout and l2-regularizations. The length of the elements in the radiomics layer was denoted as 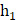, while that of clinical features was denoted as 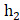, and the second layer was denoted as 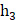. The implementation of this network segment was carried out using the PyTorch framework. For univariate analysis, we employed the scikit-learn package^22^ in Python to compare differences between groups using the t-test. In our experiments, we set 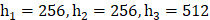. Considering the potential instability of neural networks, we conducted 20 iterations of model testing and obtained averaged metrics along with confidence intervals. For each experiment, the data was divided into three cohorts, with 40% used for training, 10% for validation, and 50% for testing. Network parameters were reset for each test. MVI and not-MVI data were randomly sampled and distributed across these cohorts, ensuring nearly equal ratios of MVI and not-MVI in all three cohorts.

### Subset Analysis on Tumor Size

The test cohort was stratified into distinct subsets based on the largest tumor diameter, as smaller tumors exhibited a decreased likelihood of demonstrating MVI. Small HCC (SHCC) was defined as either a single HCC nodule with a diameter of ≤5 cm or no more than three HCC nodules with maximum diameters of ≤3 cm^23^. A single HCC nodule measuring less than 3 cm was considered indicative of early-stage HCC, based on the Barcelona Clinic Liver Cancer stage (BCLC) criteria^24^. Consequently, the patients were categorized into three groups: a group with tumors measuring less than 3 cm, a group with tumors measuring between 3 cm and 5 cm, and a group with tumors measuring greater than 5 cm. Tumors within the intermediate size range of 3 cm to 5 cm posed a challenge as the size criterion had limited utility in this group.

### Metrics

This study employed various evaluation metrics, including accuracy (ACC), area under the receiver operating characteristic curve (AUC), specificity (SPE), and sensitivity (SEN). ACC, SPE, and SEN represented the ratios of correct predictions, computed on the entire sample, positive samples, and negative samples, respectively. AUC served as a comprehensive metric for evaluating the classification system, with higher values indicating superior recognition of both positive and negative samples. The final metrics for the model were derived from the test cohort, while the hyper-parameters were selected based on the metrics obtained from the validation cohorts. Consequently, the results section will also present the validation metrics to provide a comprehensive assessment of the model's performance.

## Results

### Univariate Analysis

Our study conducted a comprehensive univariate analysis to identify clinical features that exhibited statistically significant differences between two groups. The analysis revealed that seven clinical features, namely tumor size, aspartate aminotransferase, gamma-glutamyl transferase (GGT), total protein, lymphocyte ratio, plasma fibrinogen, and HBcAb, demonstrated significant differences between the groups (p<0.01) (Table S1).

### Multi-sequence Registration and Segmentation

This study presents an evaluation of the registration performance in HCC imaging, employing manually annotated HCC-related areas. The registration performance was quantified by calculating the Dice index between the pre-contrast T1-WI and the transformed T1-WI (hepatobiliary phase) as well as T2-WI segmentations. This metric provided a measure of the alignment accuracy achieved by the transformation process in aligning the HCC-related areas. Furthermore, segmentation performance was assessed using the Dice index, which measured the agreement between the predicted segmentations and the annotated ground truth. For the test dataset, the Dice index was determined to be 0.762 (Table S2).

### Multi- sequence Classification

**Figure 2:**
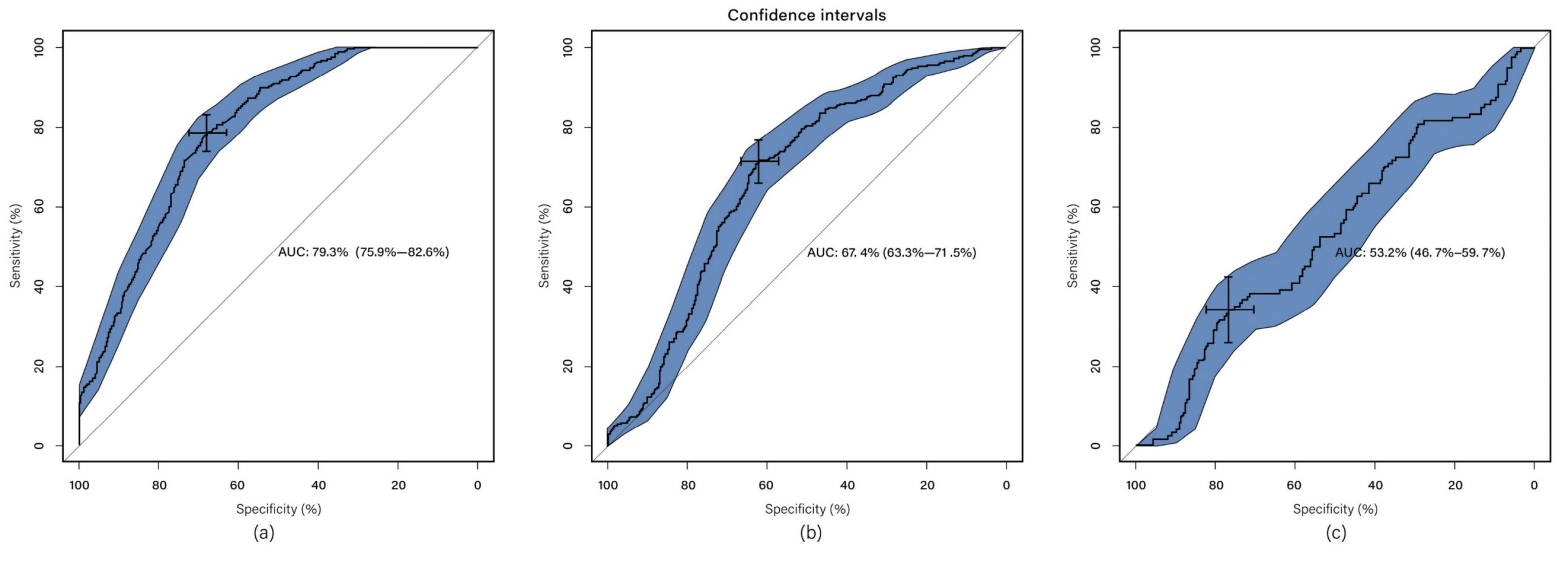
ROC curve for automated method; (a) clinical and radiomics features; (b) radiomics features (without clinical features); (c) clinical features (without radiomics features). The blue area is 95% CI.

**Figure 3:**
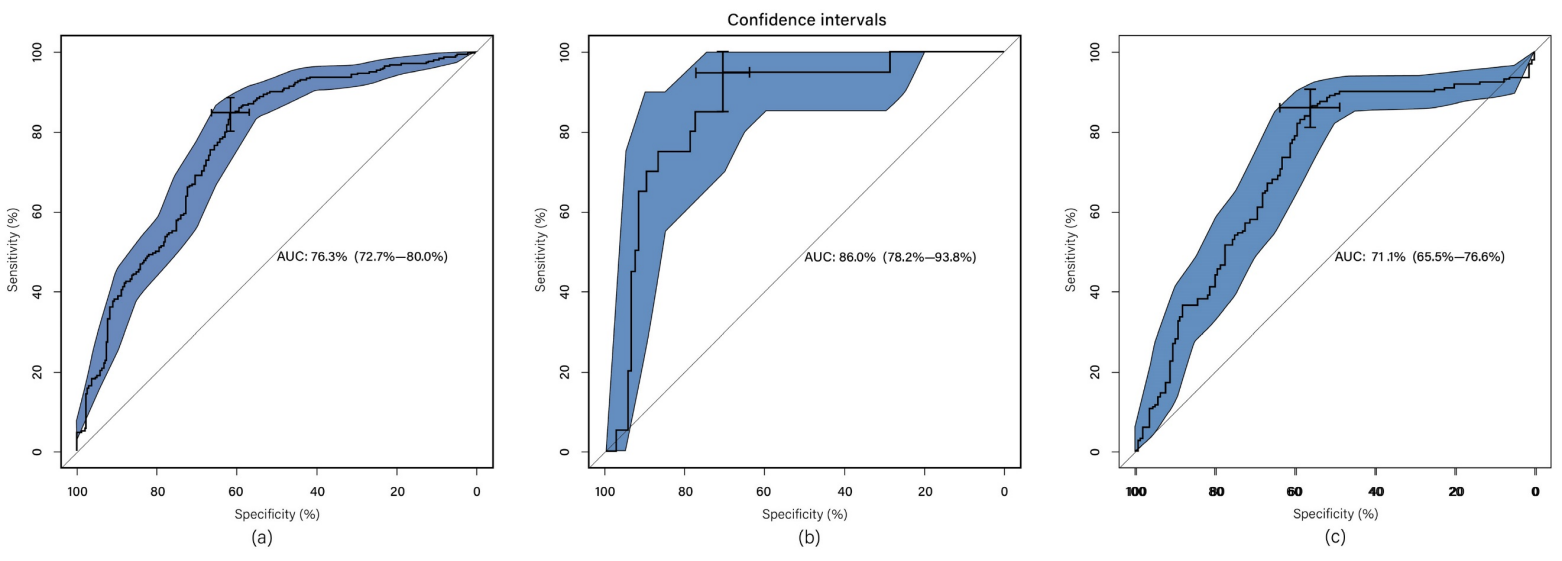
ROC curve for proposed method in subset groups; (a) <3cm group; (b) 3-5cm group; (c) >5cm group. The blue area is 95% CI.

The classification system incorporates radiomics features extracted from radiomics feature extraction and a set of seven clinical features, thus referred to as multi-modal classification. The results demonstrate that utilizing all imaging sequences yields superior performance compared to any individual sequence (Table 1). Furthermore, the results indicate that the automatic system outperforms the manual-assisted method (Table 2). The proposed method integrates clinical and radiomics features and serves as an automatic segmentation system. Evaluation against other methods shows that the proposed approach achieves an AUC of 0.794 ± 0.033, surpassing all other methods (Figure 2). Moreover, the proposed method exhibits improved performance specifically for tumors of intermediate sizes (3-5cm) with an AUC of 0.860±0.065 (Figure 3).

## Discussion

MVI is recognized as a significant risk factor for postoperative recurrence of HCC. Therefore, the accurate preoperative prediction of MVI assumes paramount importance in guiding the selection of appropriate surgical candidates and liver transplant recipients. In this context, the integration of computer-aided diagnostic technology and imaging holds immense potential as an analytical tool. This approach combines a range of data mining algorithms and statistical analysis tools with high-throughput imaging functions to derive predictive information.

Previous investigations have examined the applicability of deep learning approaches in the prediction of MVI using MRI^15^, ^16^. However, these studies have encountered challenges stemming from the limited number of available cases, posing difficulties in training deep neural networks and requiring manual delineation of tumor regions as a prerequisite for network training. In our current study, we have overcome these challenges by incorporating a considerably large number.

**Figure 4:**
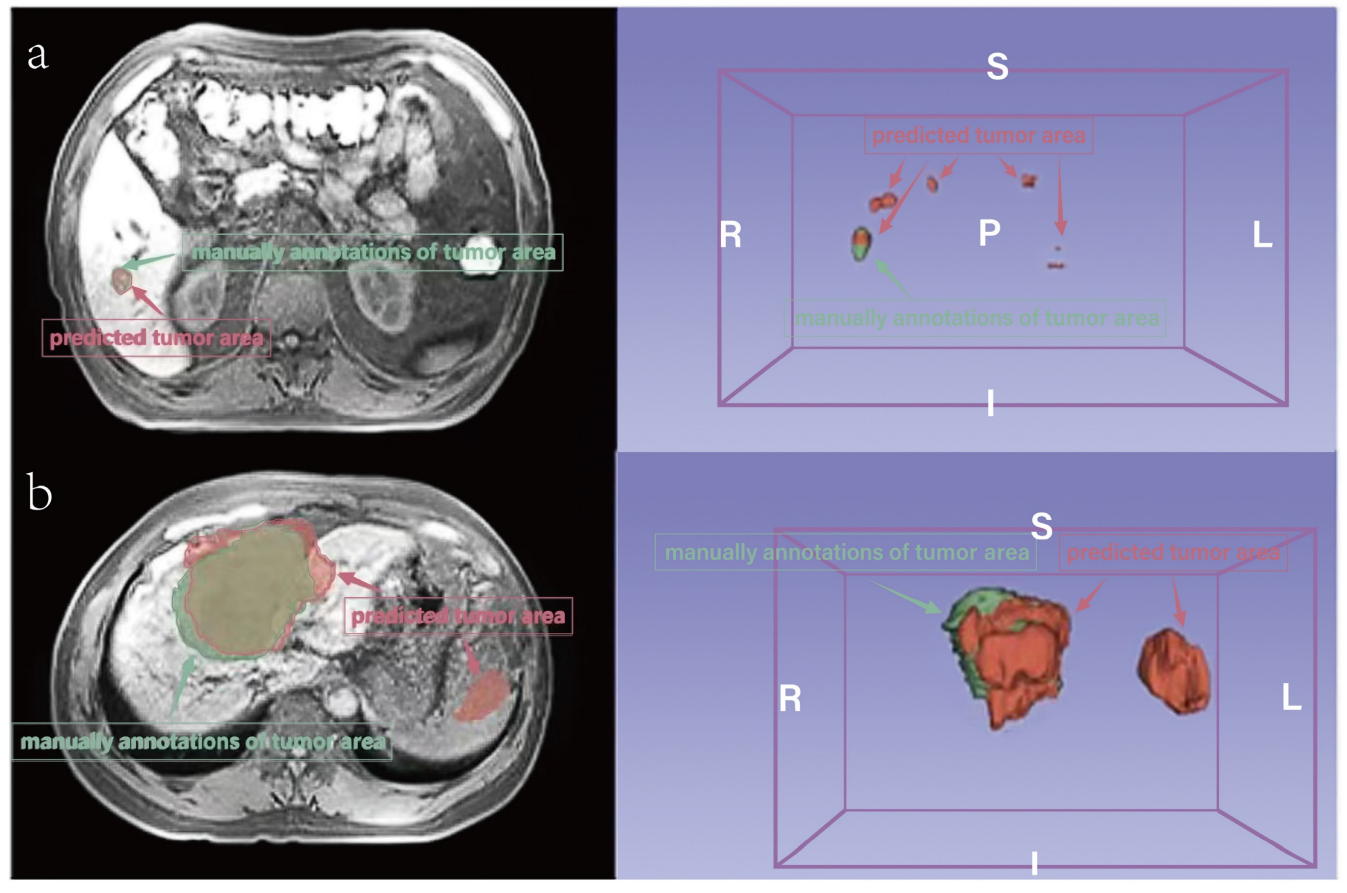
Comparison of prediction and manually annotated segmentation of HCC-related areas. a. An example with a small area (not MVI). b. An example with a large area (MVI). The red area means predicted tumor area and the green means manually annotations of tumor area.

Additionally, deep neural networks were utilized in our study to perform segmentation of HCC-related areas, replacing conventional manual methods, thereby significantly reducing labor and time requirements. While the automatic segmentations may not exhibit perfect concordance with manual annotations, the radiomics features derived from these segmentations demonstrate comparable performance. This phenomenon stems from the inherent characteristics of the target areas, which are often small and prone to occasional offsetting or mismatching when compared to the ground-truth, leading to lower Dice scores (Figure 4). Future research endeavors should consider the inclusion of larger sample sizes to facilitate improved performance and robustness of automatic segmentation techniques.

In previous studies, deep learning models for MVI prediction have primarily relied on training with imaging features alone. However, it is important to consider that certain clinical features are closely associated with HCC development and MVI prediction. Zhao *et al.*^25^ reported on predictors of MVI before surgery for multifocal liver cancer. They found that higher GGT levels, tumor diameter >8 cm, and tumor number >3 were the preoperative predictors in multifocal liver cancer patients. Another recent study involving 165 patients with HCC revealed that aspartate aminotransferase levels and lymphocyte ratios independently predicted MVI^26^. Hepatitis B viral infection is a major risk factor for cirrhosis and hepatocellular carcinoma^27^. HBV leads to increased invasiveness of hepatocellular carcinoma, and HBV may play an important role in initiating the development of MVI^28^. In our investigation, we observed elevated levels of 7 clinical features in the MVI group compared to the non-MVI group. We evaluated the predictive performance of clinical features, radiographic features, and their fusion using automated methods. Our findings indicate that the integration of clinical and radiographic features through automated methods yields superior predictive performance and merits further consideration.

The management of HCC is contingent upon factors such as tumor size, the severity of liver disease, and vascular invasion. Liver transplantation eligibility criteria encompass the following: single tumor≤5cm or multiple tumors diameter≤3cm; No evidence of vascular invasion and regional lymph node or distant metastasis (Milan criteria)^29^. Additionally, the prognosis of liver transplantation and hepatectomy is closely linked to tumor size and the presence of MVI^5^, ^6^. Moreover, tumor size is also a contributing factor to MVI^25^. It is evident that smaller HCC tumors exhibit a reduced likelihood of MVI, whereas larger tumors display an increased likelihood of MVI due to their ability to invade adjacent vessels. Consequently, preoperative prediction of MVI in patients with medium-sized tumors poses a challenge. In our study, we employed a proposed method across different size groups and observed superior performance in the medium-sized group compared to others. These findings validate the efficacy of our system in accurately identifying MVI, particularly within the medium-size tumor group (3-5cm). Based on our survey, limited research has focused on cases within this challenging group. We aspire to gather more cases in this particular cohort in the future to address this issue comprehensively.

We have undertaken an end-to-end deep learning approach, devoid of radiomics, whereby images or volumes are inputted to generate predictions. Nevertheless, both the 2-D image method and the 3-D volume method proved unsuccessful in our experiments. We attempted to replicate the approach employed by Wu^15^, involving the masking of HCC areas or the cropping of their bounding boxes, yet the network still did not yield satisfactory results. These attempts resulted in accuracies ranging from 52% to 66%. We postulated that the CNN struggles to discern valuable features from the images due to the relatively small size of our dataset in comparison to conventional natural images. Conversely, when employing radiomics to extract features, the deep neural network can effectively learn parameters for feature processing and generate predictions.

In future research, we anticipate that augmenting the dataset for this deep learning-based approach will lead to improved outcomes. Our findings demonstrate the efficacy of the experiments in predicting MVI using the radiomics-based method, providing reliable performance. However, the deep learning-based method necessitates a larger dataset for comprehensive evaluation. Furthermore, despite analyzing performance across different size groups, additional data is required to derive more robust conclusions. For future investigations, we hold the belief that the deep learning-based method has the potential to achieve superior results.

## Conclusions

According to our investigation, we compiled a comprehensive dataset consisting of multi-sequence MRI scans and clinical data obtained from 420 cases of HCC. In this research, we introduce a fully automated system designed for the preoperative MRI-based prediction of MVI in HCC. Our experimental findings demonstrate that the integration of all available sequences and clinical features leads to the attainment of optimal performance. Remarkably, even within a subgroup characterized by tumor sizes ranging from 3 to 5cm, the system maintains satisfactory performance levels, despite the heightened challenges and clinical importance associated with this particular subgroup.

## Supplementary Material

Supplementary tables.

## Figures and Tables

**Table 1:** Single sequence results and combined sequences results

Sequence	Validation				Test			
	ACC	AUC	SEN	SPE	ACC	AUC	SEN	SPE
DWI	0.790±0.087	0.924±0.073	0.713±0.187	0.857±0.096	0.693±0.03	0.749±0.018	0.242±0.087	0.986±0.023
T1-WI (pre-contrast)	0.767±0.107	0.822±0.137	0.431±0.222	0.97±0.026	0.645±0.062	0.670±0.055	0.362±0.239	0.881±0.105
T1-WI (arterial phase)	0.771±0.128	0.815±0.176	0.643±0.259	0.866±0.069	0.671±0.03	0.767±0.094	0.231±0.101	0.957±0.017
T1-WI (portal phase)	0.867±0.059	0.917±0.068	0.726±0.102	0.959±0.054	0.610±0.034	0.779±0.031	1.00±0.0	0.323±0.059
T1-WI (hepatobiliary phase)	0.810±0.075	0.910±0.092	0.677±0.11	0.898±0.135	0.519±0.019	0.775±0.011	0.962±0.033	0.151±0.049
T2-WI	0.786±0.064	0.856±0.091	0.746±0.138	0.837±0.095	0.593±0.134	0.778±0.058	0.857±0.302	0.509±0.251
All ^†^	0.793±0.134	0.882±0.058	0.857±0.302	0.509±0.251	0.689±0.127	**0.794**±**0.033**	0.900±0.138	0.536±0.091

All results here use proposed parameter setting. **^†^** means the proposed sequence setting. The value after ±is the range of 95% CI.

**Table 2:** Multi-model classification results using all sequences compared with different settings

Seg.	Radiomics	Clinical	ACC	AUC	SPE	SEN
Manual	√	√	0.723±0.069	0.797±0.074	0.621±0.222	0.828±0.091
Manual	√	×	0.771±0.028	0.703±0.122	0.782±0.132	0.827±0.059
Auto	√	×	0.638±0.094	0.674±0.057	0.762±0.451	0.408±0.112
Auto†	√	√	0.689±0.127	**0.794±0.033**	0.900±0.138	0.536±0.091
×		√	0.555±0.057	0.535±0.033	0.383±0.125	0.624±0.075

**^†^**is the proposed parameter setting. Seg. means segmentation method. The value after ±is the range of 95% CI.

## Abbreviations

MVI: microvascular invasion
MRI: magnetic resonance imaging
HCC: hepatocellular carcinoma
AFP: α-fetoprotein
CEA: carcinoma embryonic antigen
GGT: gamma-glutamyl transferase
