# Supplementary material for: Deep Neural Network and Radiomics-based Magnetic Resonance Imaging System for Predicting Microvascular Invasion in Hepatocellular Carcinoma
Source: J Cancer. 2024 Oct 14;15(19):6223–31. doi: 10.7150/jca.93712 (PMC11540505; doi:10.7150/jca.93712)
Supplement: Supplementary file 1 — Supplementary tables. [file jcav15p6223s1.pdf]

Table S1 Univariate Results for clinical features

	not MVI group	MVI group	P-value
Age(year)	59.6±11.5	58.8±12.2	0.486
Gender			0.083
Male	183(65.4%)	105(75.0%)	
Female	97(34.6%)	35(25.0%)	
Height(cm)	169.2±5.6	169.3±6.1	0.960
Weight(kg)	71.5±11.2	70.4±12.4	0.668
Number of tumors	1.8±1.6	2.1±2.3	0.062
Size of tumor	3.9±2.4	5.8±3.4	**＜0.001**
Liver function			
ALT (U/L)	35.0±33.7	39.1±27.7	0.185
AST (U/L)	30.8±21.1	37.6±25.6	**0.007**
GGT (U/L)	71.7±90.5	109.1±132.2	**0.003**
ALP (U/L)	82.8±47.6	92.7±45.2	0.039
Total protein (g/L)	68.7±8.7	71.8±5.0	**＜0.001**
Serum albumin (g/L)	41.6±6.2	42.2±4.8	0.297
TBIL (umol/L)	16.6±18.3	17.3±18.3	0.716
DBIL (umol/L)	6.4±14.2	8.3±16.6	0.245
Blood routine examination			
RBC (*10^{12}/L)	4.5±0.7	4.6±0.6	0.327
WBC (*10^{9}/L)	5.7±2.0	73.4±789.5	0.314
BPC (*10^{9}/L)	172.8±76.4	178.5±71.8	0.450
Hemoglobin (g/L)	142.1±20.4	142.1±23.2	0.973
Lymphocyte ratio	0.31±0.09	0.28±0.11	**0.004**
Neutrophil ratio	0.6±0.1	0.6±0.4	0.065
Monocyte ratio	0.1±0.0	0.1±0.0	0.617
Basophil ratio	0.006±0.006	0.006±0.003	0.215
Eosinophil ratio	0.02±0.01	0.02±0.02	0.789
Coagulation function test			
Plasma D dimer (ug/ml）	0.4±0.7	0.9±4.4	0.175
Plasma fibrinogen (g/L)	2.9±0.9	3.3±1.1	**＜0.001**
Prothrombin time (s)	13.7±2.1	13.7±1.2	0.747
Activated partial thromboplastin time (s)	36.4±4.8	37.0±4.2	0.176
Thrombin time (s)	17.7±12.0	16.8±1.4	0.264
International normalized ratio	1.1±0.1	1.1±0.1	0.603
Prothrombin time activity (%)	92.2±16.0	93.9±15.0	0.292
Hepatitis screening			
HBsAg			0.054
positive	174(62.1%)	100(71.4%)	
negative	106(37.9%)	40(28.6%)	
HbcAb			**0.003**
positive	237(84.6%)	131(93.6%)	
negative	43(15.4%)	9(6.4%)	

HbeAg			0.379
positive	46(16.4%)	28(20.0%)	
negative	234(83.6%)	112(80.0%)	
HBeAb			0.088
positive	177(63.2%)	100(71.4%)	
negative	103(36.8%)	40(28.6%)	
HbsAb			0.752
positive	74(26.4%)	35(25.0%)	
negative	206(73.6%)	105(75.0%)	
HCVAb			0.666
positive	8(2.9%)	3(2.1%)	
negative	272(97.1%)	137(97.9%)	
Serum tumor markers			
AFP (ng/ml)	733.0±2828.2	1712.5±5526.5	0.051
CEA(ng/ml)	5.4±27.0	2.8±2.1	0.109
CA125 (u/ml)	15.6±26.7	20.1±35.0	0.186
CA15-3 (u/ml)	10.6±8.1	10.8±7.5	0.895
CA724 (u/ml)	3.3±5.0	3.4±5.3	0.863
CA19-9 (u/ml)	46.8±210.1	59.3±327.4	0.681

Table S2 Registration performance

Transform type	Dice Before Registration	Dice After Registration
Rigid	0.534	0.762
Non-Rigid		0.496
